# Partial Freezing Enables Functional Preservation of Kidney Grafts for up to 10 Days

**DOI:** 10.21203/rs.3.rs-7820837/v1

**Published:** 2026-01-14

**Authors:** Korkut Uygun, McLean Taggart, Madeeha Hassan, Arnaud Lyon, O. Sila Ozgur, Christopher Taveras, Mohammadreza Mojoudi, Thomas Agius, Sophie Van Tulder, Christina Zheng, Michael Macarthur, James Markmann, Heidi Yeh, Shannon Tessier, Mehmet Toner, Alban Longchamp

**Affiliations:** Massachusetts General Hospital, Harvard Medical School; Massachusetts General Hospital - Harvard Medical School; Massachusetts General Hospital, Harvard Medical School; Massachusetts General Hospital & Harvard Medical School; Massachusetts General Hospital, Harvard Medical School; Massachusetts General Hospital, Harvard Medical School; Massachusetts General Hospital, Harvard Medical School; ETH Zurich; Massachusetts General Hospital - Harvard Medical School; Princeton University; Princeton University; Massachusetts General Hospital - Harvard Medical School; Massachusetts General Hospital, Harvard Medical School; Massachusetts General Hospital, Harvard Medical School

## Abstract

Kidney transplantation is critically limited by the shortage of available donor organs, exacerbated by the discarding of viable organs due to insufficient allocation time. Here, we demonstrate the application of nature-inspired partial freezing for long-term storage of kidney grafts. We show that, after 10 days of storage, partially frozen swine and human kidneys retain improved function compared to those preserved using traditional static cold storage, as assessed via simulated transplantation.

Kidney transplantation leads to greatly reduced mortality and improved quality of life when compared to long-term dialysis for end-stage renal disease ^1, 2^; however, access is greatly limited by the number of available donor organs. In 2022, 26,000 kidney transplants were performed, while 44,000 new adult candidates were added to the waiting list, a discrepancy of 18,000 ^3^. When accounting for individuals not added to the waiting list due to disqualifying criteria, it has been estimated that this discrepancy could potentially increase 10-fold ^4, 5^. Approximately 25% of procured kidneys are discarded for various reasons, including biopsy status, kidney donor profile index (KDPI), and cold ischemic time (CIT) ^3, 6^. Most clinics limit their kidney usage to those with less than 24 hours of CIT, leading to excessive discard ^7, 8^. With improved storage techniques, the period of viable storage could be markedly expanded, enabling improved allocation ^4, 9^. Furthermore, in the context of xenotransplantation, storage periods over a week in length will facilitate global allocation of pig kidneys, enabling a few, centralized facilities for production ^10^.

Active preservation approaches, such as machine perfusion (MP), have recently demonstrated successful long-term storage up to 4 days; however, their clinical application is technically challenging and costly, and clinical trials have failed to demonstrate improved graft function for kidneys ^11–14^. Several cryopreservation approaches, which aim to leverage temperature-mediated metabolic suppression to prolong storage, have been developed; however, they are limited by stability, storage period, and/or technological complexity ^15–22^. To overcome such limitations, we have recently developed partial freezing (PF) for livers, a nature-inspired cryopreservation approach, taking inspiration from the *Rana Sylvatica* wood frog ^23, 24^. PF leverages machine perfusion to load organs with low concentrations of cryoprotective agents (CPAs) ^25, 26^. This approach has been shown to enable prolonged storage in rat livers at −15°C up to 10 days ^25, 27^.

In this work, we provide the first literature demonstration of pig and human kidney cryopreservation over a period of one week, using the PF methodology. Briefly, kidneys were gradually loaded with 3-O-methyl-D-glucopyranose (3-OMG; a glucose analog and metabolic depressor), SnoMax (a potent ice nucleating agent (INA) which allows spatial and temporal control of ice formation), polyethylene glycol 35k (PEG; a membrane stabilizer), trehalose (an extracellular CPA), and propylene glycol (PG; an intracellular CPA). This leads to a progressive cellular dehydration, thereby restricting ice formation to the endothelium and not inside the intracellular space, which is where the term partial freezing originates from. Using a simulated transplantation model, the viability of pig and human kidneys stored using PF and static cold storage (SCS) was compared. We demonstrate that kidney PF leads to improved organ function following long-term storage in both pig and human kidneys.

Kidneys were procured from dead by brain death (DBD) pigs after < 20 minutes of warm ischemic time (WIT). They then underwent the PF loading process, whereby increasing concentrations of CPAs were sequentially loaded while temperature was progressively decreased to minimize cytotoxicity (Fig. 1a). CPA loading leads to a progressive cellular dehydration, thereby restricting ice formation to the endothelium outside the intracellular space (Fig. 1b). Kidneys were then stored using either PF or traditional SCS for 3- or 10-days. Following storage, CPAs were unloaded from PF kidneys, and they underwent a 3-hour recovery phase using subnormothermic MP (SNMP) with an oxygenized, acellular perfusate at 21°C. No difference in perfusion parameters was observed during this phase between kidneys stored for 3- and 10-days (**Fig. S1a-S1c**). Perfusate lactate, potassium concentration, and the creatinine clearance rate were similar in all groups (**Fig. S1d-S1e**). No difference in histological damage was observed as assessed by a modified version of the Goujon score^28^ (**Fig. S1j-S1k**). At the end of both PF and SCS, kidneys underwent a simulated transplantation, whereby they were perfused with a 20% whole blood solution at 37°C using MP to replicate the reperfusion process happening at the time of transplantation. As a control, we also introduced a group of kidneys, labeled “Fresh,” which underwent simulated transplantation immediately following procurement. Macroscopically, PF kidneys exhibited more homogenous perfusion compared to those that underwent SCS, demonstrating a closer resemblance to fresh kidneys (Fig. 1c).

Tracking perfusion parameters during machine perfusion reveals key insights into organ function and health^29^. Arterial resistance is a measurement of vascular function, and increased resistance is associated with allograft dysfunction^30^. Resistance was significantly lower in 3-day PF and 10-day PF kidneys compared to SCS, demonstrating improved perfusion (Fig. 1d). AUC analysis of resistance showed a significant reduction in kidneys that underwent PF, with no difference between 3-day and 10-day. Immediately after storage, transmission electron microscopy (TEM) of kidneys revealed fragmented glomerular endothelial cells in SCS kidneys, whereas PF kidneys retained fenestrated endothelial cells with intact tight basement membranes. Following simulated transplantation, podocytes in SCS kidneys were fragmented and atypical, while 10-day PF retained a more standard, recognizable podocyte structure, supporting improved endothelial integrity (**Fig. S2**). Weight gain, a measure of interstitial edema, was similarly reduced in both PF groups compared to SCS, though non-significantly (Fig. 1f). Both venous potassium and venous lactate, markers of cellular injury, trended lower in PF kidneys compared to SCS (Fig. 1g/h), showing significant reductions at the terminal time point (**Fig. S3a/b**). Creatinine clearance rates were not statistically different across groups, although median values were lowest in SCS groups (Fig. 1i). Urine production, a key factor in kidney perfusion viability assessment^31^, was retained in all PF kidneys (Fig. 1j), with the difference reaching clinical significance in 3-day PF compared to 3-day SCS (Fig. 1k). PF kidneys produced more urine than SCS kidneys at matched storage durations, even though important individual variations were observed. Lack of urine production in fresh kidneys is likely an experimental artifact caused by imperfect perfusion.

At the end of simulated transplantation, no difference in histological scoring (where 25 is the greatest degree of injury, and 0 is no injury ^28, 32^) was seen between 3-day SCS and 3-day PF; however, 10D PF was nonsignificantly reduced by 22.6% compared to 10-day SCS. Excitingly, no difference was seen between fresh and 10-day PF kidneys. Interestingly, in 10-day PF kidneys, scoring was found to increase over the storage period, with no significant elevation in scoring occurring from post-storage to the end of perfusion, indicating no exacerbation of injury following storage (**Fig. S4**). The opposite trend was observed in 10-day SCS kidneys, where no elevation in scoring was observed during storage, but a large increase in injury was seen from post-storage to the end of perfusion. This trend was not seen in 3-day PF kidneys. This indicates that long-term partial freezing dynamics may be minimally injurious, while dampening the impact of ischemia-reperfusion injury. 30-day PF was also attempted; however, elevated injury markers (**Fig. S5**) and dramatic morphologic injury (**Fig. S6**) indicate that the current protocol is insufficient for such storage times.

To advance kidney partial freezing towards clinically relevant models, we applied the protocol to discarded human grafts. To control for the wide range of quality in grafts rejected for transplantation (**Table S1**), a 3-hour recovery phase was added before storage, enabling direct comparison of the pre- and post-storage SNMP (**Fig. S7**). Macroscopically, no difference in gross morphology was observed at the end of SNMP before and after storage (Fig. 2a). By 60 minutes of SNMP perfusion, resistance remained steady (Fig. 2b), with no difference in terminal resistance before and after storage (Fig. 2c). Additionally, venous lactate and potassium were steady (Fig. 2d/e), with no difference in terminal values (**Fig. S8a/b**). Three out of four 3-day PF kidneys and two of three 10-day PF kidneys produced no urine before storage; however, all kidneys demonstrated urine production after storage (Fig. 2f). Kidneys stored for 10 days demonstrated significantly elevated urine production, potentially indicating leakage (**Fig. S8c**). The post-storage creatinine clearance rates were 50% and 57.5% for 3-day PF and 10-day PF, respectively, compared to pre-storage, with no significant difference in retention observed between the storage periods (**Fig. S8d**). Tissue microstructure was well-maintained throughout the PF process (Fig. 2h). No difference was seen in histological scoring except for immediately after storage, indicating that the longer periods of storage may lead to a greater degree of storage-mediated injury (**Fig. S9a**); however, no difference was observed at the end of the SNMP post phase. Similarly, no difference was observed in the change in histological scoring between time-points (**Fig. S9b**).

Human kidneys also underwent simulated transplantation following SNMP recovery. Macroscopically, 3-day PF kidneys showed more homogenous perfusion, with an absence of dark patches which would indicate poor perfusion, whereas 10-day PF kidneys showed some regional, heterogenous darkening (Fig. 2i). Arterial resistance was steady throughout perfusion for both storage periods (Fig. 2j), and no difference was seen in terminal resistance (**Fig. S8e**). No difference was observed between 3-day PF and 10-day PF kidneys in creatinine clearance rate (Fig. 2k). All kidneys produced urine during simulated transplantation (Fig. 2l), and no difference in total urine production was observed (**Fig. S8f**). A quality assessment score was calculated as previously described^13^: 3/4 3-day PF and 1/3 10-day PF kidneys had a score of 3 or less (Fig. 2m); such scoring has been previously described as potentially transplantable^31^. Histologically, tissue microstructure was consistent between 3-day PF and 10-day PF kidneys (Fig. 2n). No difference was observed between terminal histological scoring (Fig. 2o), while 4/4 3-day PF and 2/3 10-day PF had an increase between 0 to 3 points (**Fig. S9c**). Metabolomics was performed throughout the PF process, showing an increase in TCA cycle and glycolysis metabolites during recovery (**Fig. S10**). This increase was found in both 3- and 10-day PF, showing no storage-time dependent difference.

It must be noted that the performance of human kidneys in this study is significantly impacted by the quality of the kidneys before experimentation. All kidneys were received from DBD donors, which are typically transplanted, meaning all kidneys we obtained were rejected for non-trivial reasons; the kidneys used in this study had a median cold time of 18.9 hours and a median KDPI of 95 (**Table S1**). For example, the donor of one pair of kidneys was diagnosed with CKD stage 3b, indicating > 50% loss in graft function compared to a healthy kidney^33^. The relatively poor quality of kidneys used in this study could explain why the human grafts appeared to function worse following extended storage compared to the swine counterparts.

Access to kidney transplantation is critically bottlenecked by an insufficient supply of donor grafts. Donor human kidney utilization can be improved through the application of prolonged organ storage, enabling better donor cross-matching and the development of international allocation networks. Similarly, long-term storage would allow the distribution of gene-edited organs globally from a limited number of specialized farms for xenotransplantation. In this study, we demonstrate the first application of partial freezing to both pig and human kidneys, showing that the technique results in improved organ function following prolonged storage periods for up to 10 days. According to clinically used viability criteria, the partially frozen kidneys would be considered transplantable, indicating successful preservation for the purposes of this preclinical study. Technically, this approach builds on standard machine perfusion platforms and easy-to-access storage containers, and offers specific advantages compared to other techniques, which are thermodynamically unstable^34, 35^ or technologically complex^20, 36^.

This paper demonstrates translation of partial freezing protocol from previous liver studies^25–27^ to kidneys, demonstrating a first-in-literature in subzero preservation of pig and human kidneys. Given that the PF protocol was developed initially for liver preservation, we expect that with cell-type-specific CPA optimization, these results may be further optimized. A critical limitation of the study is that simulated transplantation with MP does not fully replicate the complex in vivo environment of a recipient organism, including immune responses, hormonal signaling, and systemic hemodynamic regulation. The advent of week-long kidney storage could represent a paradigm shift in the field of transplantation and may enable the field to push beyond scarcity, enabling on-demand transplantation for those who need it. We also demonstrate potential for extension up to a month, which would be transformative for organ allocation, enabling global allocation or transport of organs via more cost-efficient land or sea routes, particularly relevant for xenotransplantation purposes.

## Methods

### Experimental Design

Experiments using pig kidneys were divided into six groups: Fresh (N=5), which underwent simulated transplantation within 2 hours of procurement; 3-day (N=4) and 10-day (N=5) SCS, which were kept at 4°C for the respective storage period before simulated transplantation; and 3-day (N=4), 10-day (N=6), and 30-day (N=5) PF, which were stored at −15°C following the partial freezing protocol before simulated transplantation.

Experiments using discarded human kidneys were divided into two groups: 3-day (N=4) and 10-day (N=3) PF, which were stored at −15°C after undergoing the modified human kidney partial freezing protocol before simulated transplantation.

### Static Cold Storage

Kidneys stored using static cold storage were flushed with 100 mL of University of Wisconsin solution (UW) after back-tabling. They were then submerged in 200 mL of UW and placed in a temperature-controlled refrigerator to maintain a constant temperature of 4°C.

### Kidney Procurement

Pig kidney experiments were approved by the Institutional Animal Care and Use Committee (IACUC) of Massachusetts General Hospital (Boston, MA, USA; protocol # 2023N000042). All experiments were performed according to the requisite guidelines and regulations.

Yorkshire pigs (40 kg, mixed sex) were obtained one week before experimentation to allow for acclimatization. Pigs were sedated with a mixture of atropine (0.4 mg/kg), telazol (4.4 mg/kg), and xylazine (2.2 mg/kg), as well as an IV bolus of propofol (0.16 – 0.33 mg/kg). Anesthesia was maintained with continuous isoflurane (3–5%) inhalation and IV fentanyl (5 – 20 ug/kg/h) as needed. Following a heart procurement^37^, the aorta was cannulated, and a bolus of heparin (100 U/kg) was administered. Immediately after the cross-clamp was applied, a systemic flush was performed through the aorta. The kidneys were then exposed and dissected from connecting tissue, after which the renal vein and artery were dissected and the kidneys removed from the abdomen. Immediately after explant, the renal artery was cannulated with a 14G cannula, which was secured with 2–0 Prolene and flushed with 500 mL of LR. The kidneys were then backtabled; the remaining connective tissue was removed from the kidney surface, the vasculature was skeletonized, and the ureter was cannulated with a 14G cannula.

### Partial Freezing Protocol

The kidney partial freezing protocol was adapted from previous works^25–27^. Solution compositions are as outlined (**Table S2**). Partial freezing was performed on a homemade, temperature-controlled system, enabling time-resolved control of perfusate. For pig kidneys, the system was set to 21°C and loaded with loading solution. Kidneys were flushed with > 100 mL of LR and attached to the system at a flow rate of 10 mL/min, which was increased by 10 mL every 5 minutes until reaching an arterial pressure of 55 mmHg. After 40 minutes, the solution was gradually changed to storage 1 solution (25 mL, 10% increments), and the temperature was set to 4°C. After reaching 100% S1, the perfusion was continued for 40 minutes; the temperature takes approximately 20 minutes to reach 4°C. Throughout this process, the flow is gradually reduced to keep the pressure below 55 mmHg. A gradual change was then performed to switch to storage solution 2. This phase was run for 30 minutes, keeping the pressure the same as the terminal pressure for S1. After 30 minutes of perfusion, the kidney was removed from the system and submerged in 200 mL of S2 in a plastic bag. The bags were then submerged under 50% PG solution at −15°C to maintain constant temperature.

Following storage, the kidneys underwent an unloading and recovery phase. The perfusion system was loaded with thawing solution at 4°C. Kidneys were removed from submersion, and the partially frozen solution was removed from the kidney surface. The kidneys were then submerged in 100 mL of thawing solution at 37°C for 5 minutes, or until the core ice of the kidney was thawed. They were then attached to the system at 5 mL/min and allowed to acclimate for 5 minutes, after which the flow rate was increased by 5 mL/min every 5 minutes, maintaining pressure < 35 mmHg. After 20 minutes, the system was set to 21°C, taking approximately 20 minutes to equilibrate. After 40 minutes, the solution was gradually switched to recovery solution (25 mL, 10%) and perfused for 3 hours. Throughout this phase, the flow rate was continuously increased until reaching a pressure of 55 mmHg, which was maintained throughout. Following recovery, the kidneys underwent simulated transplantation. Biopsies were taken at the following timepoints: before perfusion (initial), immediately after pre-thaw submersion (post-thaw), and after recovery (post-SNMP), and bisected – half was flash-frozen, and the other was formalin-fixed.

For human kidneys, the following changes were made: a 3-hour pre-loading recovery phase was performed; submersion-thawing was prolonged to 10 minutes; the post-storage recovery process was preceded by a 60-minute recovery 1 phase, to gradually remove PEG; an additional biopsy was taken after pre-loading recovery. Human kidney partial freezing solutions compositions are as outlined (**Table S3**).

### Human Kidney Acquisition

This study was approved by the Massachusetts General Hospital Institutional Review Board and the New England Donor Service (NEDS; Agreement #2024A010114). All procedures were conducted in compliance with the established, aforementioned guidelines. Human kidneys that were rejected for transplant were accepted according to established criteria (**Table S4**). Kidneys were procured using established surgical techniques and either stored in UW on ice, or hypothermically perfused under hypoxic conditions. Human kidneys were backtabled following the same procedure as pig kidneys, and all cysts were kept. Before perfusion, the kidneys were flushed with > 250 mL of LR.

### Simulated Transplant

Simulated transplantation was performed using a LiverAssist device that was modified to enable one-sided perfusion while maintaining accurate temperature. Pig kidneys were perfused with 1L of William’s E-based blood solution containing 20% whole abattoir blood for 2 hours at 37°C. The pressure was maintained at 75 mmHg, a value previously optimized for kidney perfusion^38^. Every 30 minutes, 3 mL of perfusate was collected and spun down at 4000G to remove blood cells. At the end, kidneys were weighed, and 2 biopsies were taken – one was flash-frozen, and the other formalin-fixed.

For human kidneys, the same procedure was performed; however, 2L of perfusate was used, containing one unit of packed red blood cells and one unit of fresh-frozen plasma.

### Viability Assessment

Throughout perfusion, the arterial flow rate and pressure were recorded every 10 minutes, and the graft weight was measured at specific time points: before perfusion, before storage, immediately after removal from storage, after recovery, and after simulated transplantation. Arterial resistance was calculated as: $\text{Resistance}=({\text{Pressure}}_{\text{t}}/{\text{Flow rate}}_{\text{t}}/{\text{weight}}_{\text{i}})$ where t is the respective time point, and i is initial, before perfusion.

Perfusate samples were collected every 30 minutes of each phase, and blood gas analysis was performed using a Siemens RapidPoint 500. During simulated transplantation, samples were spun at 4000G for 10 minutes prior to storage at −80°C to remove blood components. Urine was collected and stored hourly. Creatinine was measured using i-STAT creatinine cartridges at 30-minute intervals. AST was measured using Piccolo Xpress Biochemical Panel Plus cartridges. The oxygen consumption rate was calculated as: $(({\text{O}}_{2,\text{inflow,t}}-{\text{O}}_{2,\text{outflow,t}})\cdot {\text{flow rate}}_{\text{t}}\cdot 0.0314)/{\text{weight}}_{\text{i}}$. Weight gain was calculated as $(({\text{weight}}_{\text{t}}-{\text{weight}}_{\text{i}})/{\text{weight}}_{\text{i}})*100$.

### Histology

Histology samples were moved from formalin to 70% ethanol after 24 hours. Tissue samples were processed by MGH Histology Molecular Pathology Core Facility. All samples were stained with hematoxylin & Eosin (H&E) and periodic acid-Schiff (PAS). Slides were then scanned using a NanoZoomer digital slide scanner for analysis. Slides were analyzed using a modified Goujon scoring method^28^, as previously described^32, 39^. Briefly, kidneys were scored on six parameters: 1) glomeruli integrity, 2) tubule dilatation, 3) brush border integrity, 4) debris in the tubules, 5) presence of vacuolizations in tubular cells, and 6) interstitial edema. Within each category, each item was graded on a scale of 0 to 3, with 0 representing “no damage” and 3 representing “extremely damaged.” Briefly, to assess glomerulus integrity, more than ten glomeruli were randomly selected from the section and assigned a score of 0 to 3 on a scale of 0 to 3. The same procedure was followed in the remaining categories. After that, the score for each category was converted to a percentage of damage. A final score between 0 and 5 was assigned based on this percentage of damage: 0 represents a percentage of damage between 0% and 15%; 1 represents a percentage of damage between 15% and 30%; 2 represents a percentage of damage between 30% and 45 percent; 3 represents a percentage of damage between 45 percent and 60%; 4 represents a percentage of damage greater than 60%; and 5 represents a percentage of damage greater than 75%. On a scale of 0 to 30, the final score was the sum of the scores for each category.

### Transmission-Electron Microscopy

Small (1 × 1 mm) tissue blocks were excised from larger specimens and fixed at room temperature in 2.0% paraformaldehyde/2.5% glutaraldehyde in 0.1 M sodium cacodylate buffer (pH 7.4), followed by overnight fixation in fresh fixative at 4 °C. Samples were rinsed in 0.1 M cacodylate buffer, post-fixed in 1% osmium tetroxide for 1 hr, rinsed again, and dehydrated through graded ethanols to 100%, then briefly in 100% propylene oxide (10 min). Tissues were infiltrated in 2:1 propylene oxide Eponate for 2 hrs, then 1:1 overnight at room temperature on a gentle rotator. The next day, samples were placed in 100% Eponate for several hours on a rocker and embedded in flat molds, polymerized at 60 °C for 24–48 hrs. Ultrathin (70 nm) sections were cut with a Leica EM UC7 ultramicrotome, mounted on formvar-coated grids, stained with 2% uranyl acetate and Reynold’s lead citrate, and imaged using a JEOL JEM 1011 TEM at 80 kV with an AMT digital imaging system.

### Metabolomics Measurements

Fluid samples (5uL) were added to 50uL of ice cold 100% methanol, vortexed and incubated on dry ice for 20 minutes. The extract was centrifuged at 23,000xg for 15 minutes at 4C 20uL of supernatant was added to 60uL of ice cold 100% methanol followed by another dry ice incubation and spin. The final supernatant was transferred to snap top microvials for LC-MS analysis.

Metabolite measurements were performed on a Thermo Q Exactive HF mass spectrometer coupled to hydrophobic interaction liquid chromatography (HILIC). A Waters XBridge BEH Amide column (150mm x 2.1mm) was used. The gradient used solvent A (95%:5% H2O:acetonitrile with 20mM ammonium acetate, pH 9.4) and solvent B (100% acetonitrile) as follows: 90% B (0.0 to 2.0 min), 90% B to 75% B (2.0 to 3.0 min), 75% B (3.0 to 7.0 min), 75% B to 70% B (7.0 to 8.0 min), 70% B (8.0 to 9.0 min), 70% B to 50% B (9.0 to 10.0 min), 50% B (10.0 to 12.0 min), 50% B to 25% B (12.0 to 13.0 min), 25% B (13.0 to 14.0 min), 25% B to 0.5% B (14.0 to 16.0 min), 0.5% B (16.0 to 20.5 min), then stayed at 90% B for 4.5 min. The flow rate was 150uL/min with an injection volume of 6uL and column temperature of 25C. Electrospray ionization (ESI) source parameters were as follows: spray voltage, 2,300 V (positive) or −2,800 V (negative); sheath gas, 35 arb; aux gas, 10 arb; sweep gas, 0.5 arb; ion transfer tube temperature, 300C; vaporizer temperature, 35C. Data acquisition was performed under a full scan polarity switching modes ranging from 70 to 1,000 m/z.

The LC-MS raw data files (.raw) were converted to mzXML format using ProteoWizard software. EL-MAVEN was used to select peaks based on an internally validated knowns list and generate an output table containing m/z, retention time and intensity for peaks. The default EL-MAVEN parameters were used for peak picking except RT shift was restricted to less than 1 minute and ppm tolerance was set to 5 ppm. Relative abundances of metabolites were visualized using R.

### Statistical Analysis

Statistical analysis and graphing were performed using GraphPad Prism 10 (version 10.4.1). Data was reported and plotted as mean ± standard error of the mean (SEM). An ordinary one-way ANOVA with Tukey’s multiple comparisons test was performed for multivariate pairwise comparisons. Comparisons between two variables were performed using an unpaired t-test. Two-sided significance was set at p < 0.05. All graphs and images were created by the authors, using photographs taken by the authors during experimentation.

## Supplementary Material

Supplementary Files

This is a list of supplementary files associated with this preprint. Click to download.

• KidneyPFNatureBiotechSupplement10.4.25.docx

## Figures and Tables

**Figure 1.**
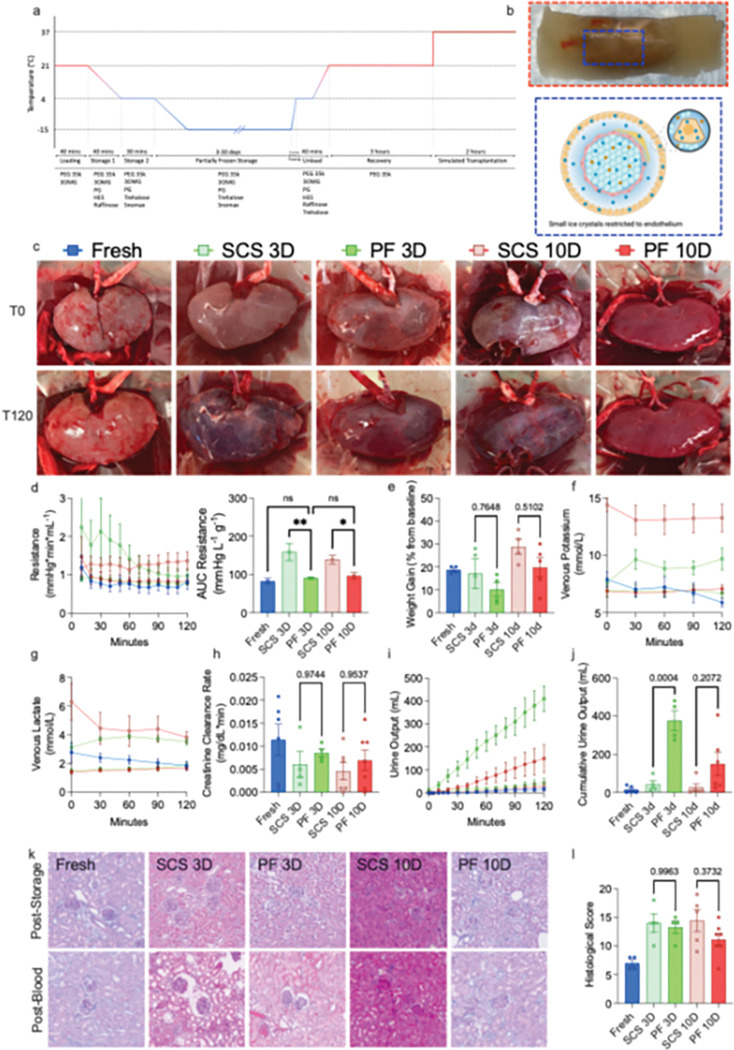
Partial Freezing Improves Pig Kidney Function Following Long-Term Storage. **a,** Schematic diagram outlining the kidney partial freezing protocol, including cryoprotectants added in each section of the protocol. **b,** Top: Picture of pig kidney immediately before thawing. Bottom: Diagram detailing hypothesized ice dynamics during partial freezing. **c,** Representative, macroscopic images of kidneys at the beginning and end of simulated transplantation. Graph detailing the **d,** Left: arterial resistance, and Right: area under the curve of resistance during simulated transplant. **f,** graph detailing kidney weight gain following simulated transplant. Graph detailing the venous **g,** potassium, and **h,** lactate throughout the simulated transplant. **i,** Graph detailing the creatinine clearance rate throughout the simulated transplant. Graph detailing the **j,** urine output and **k,** cumulative urine output throughout the simulated transplant. **l,** representative, microscopic histological images at the end of storage, and following simulated transplant. **m,** Graph detailing the histological scoring at the end of the simulated transplant. Pairwise comparison was conducted by ordinary one-way ANOVA with Tukey’s multiple comparisons test. * p < 0.05; ns, not significant

**Figure 2.**
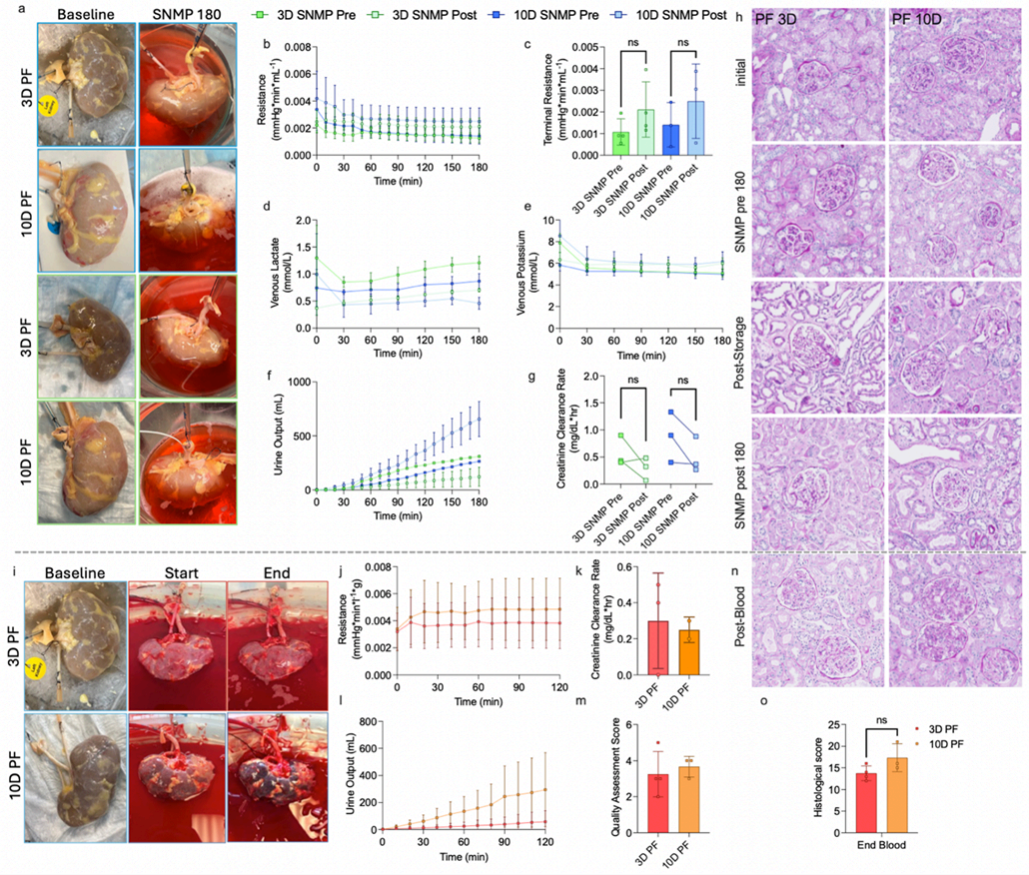
Partial Freezing Retains Kidney Function in Discarded Human Kidney Grafts. **a,** Representative images of partially frozen human kidneys before and after subnormothermic recovery. Graphs detailing perfusion parameters during subnormothermic machine perfusion, including **b,** arterial resistance, **c,** terminal arterial, **d,** venous lactate, **e,** potassium, **f,** urine output, and **g,** creatinine clearance rate. **h,** Histology stained with periodic-acid Schiff at several points throughout the partial freezing process. **i,** Representative images of human kidneys undergoing simulated transplantation. Perfusion parameters during simulated transplantation including **j,** arterial resistance, **k,** creatinine clearance rate, and **l,** urine output. **m,** Calculated quality assessment score for partially frozen grafts following simulated transplantation. **n,** Representative histology of kidneys following simulated transplantation, including **o,** histological scoring. Pairwise comparison was conducted by ordinary one-way ANOVA with Tukey’s multiple comparisons test. * p < 0.05; ns, not significant

## Data Availability

The datasets generated during and/or analyzed during the current study are available from the corresponding author on reasonable request.

## References

[1] Tonelli M (2011) Systematic review: kidney transplantation compared with dialysis in clinically relevant outcomes. Am J Transpl 11:2093–2109 10.1111/j.1600-6143.2011.03686.x 21883901

[2] Wolfe RA (1999) Comparison of mortality in all patients on dialysis, patients on dialysis awaiting transplantation, and recipients of a first cadaveric transplant. N Engl J Med 341:1725–1730 10580071 10.1056/NEJM199912023412303

[3] Lentine KL (2024) OPTN/SRTR 2022 Annual Data Report: Kidney. Am J Transpl 24:S19–S118 10.1016/j.ajt.2024.01.012 38431360

[4] Giwa S (2017) The promise of organ and tissue preservation to transform medicine. Nat Biotechnol 35:530–542 28591112 10.1038/nbt.3889 PMC5724041

[5] Ahmad FB, Cisewski JA, Anderson RN (2024) Mortality in the United States - Provisional Data, 2023. MMWR Morb Mortal Wkly Rep 73:677–681 39116025 10.15585/mmwr.mm7331a1 PMC11309370

[6] Lentine KL (2019) Variation in use of procurement biopsies and its implications for discard of deceased donor kidneys recovered for transplantation. Am J Transpl 19:2241–2251 10.1111/ajt.15325 30809941

[7] Zhao H, Alam A, Soo AP, George AJT, Ma D (2018) Ischemia-Reperfusion Injury Reduces Long Term Renal Graft Survival: Mechanism and Beyond. EBioMedicine 28, 31–42 29398595 10.1016/j.ebiom.2018.01.025 PMC5835570

[8] Malek M, Nematbakhsh M (2015) Renal ischemia/reperfusion injury; from pathophysiology to treatment. J Ren Inj Prev 4:20–27 26060833 10.12861/jrip.2015.06 PMC4459724

[9] Hotta K, Hirose T, Kawai T (2022) Clinical trials for renal allograft tolerance induction through combined hematopoietic stem cell transplantation: A narrative review. Int J Urol 29:1397–1404 36101964 10.1111/iju.15035

[10] Cutrone AM (2025) Cryopreservation Strategies to Improve Access to Organ Transplantation. Transplantation 10.1097/TP.0000000000005494 PMC13359655 40702598

[11] Kataria A, Magoon S, Makkar B, Gundroo A (2019) Machine perfusion in kidney transplantation. Curr Opin Organ Transpl 24:378–384 10.1097/MOT.0000000000000675 31205093

[12] de Haan MJA (2024) A cell-free nutrient-supplemented perfusate allows four-day ex vivo metabolic preservation of human kidneys. Nat Commun 15:3818 38740760 10.1038/s41467-024-47106-w PMC11091145

[13] Hosgood SA (2023) Normothermic machine perfusion versus static cold storage in donation after circulatory death kidney transplantation: a randomized controlled trial. Nat Med 29:1511–1519 37231075 10.1038/s41591-023-02376-7 PMC10287561

[14] Husen P (2021) Oxygenated End-Hypothermic Machine Perfusion in Expanded Criteria Donor Kidney Transplant: A Randomized Clinical Trial. JAMA Surg 156:517–525 33881456 10.1001/jamasurg.2021.0949 PMC8060886

[15] Berendsen TA (2014) Supercooling enables long-term transplantation survival following 4 days of liver preservation. Nat Med 20:790–793 24973919 10.1038/nm.3588 PMC4141719

[16] de Vries RJ (2019) Supercooling extends preservation time of human livers. Nat Biotechnol 37:1131–1136 31501557 10.1038/s41587-019-0223-y PMC6776681

[17] Powell-Palm MJ (2020) .B. Isochoric conditions enhance stability of metastable supercooled water. Appl Phys Lett 116:123702

[18] Năstase G (2023) Isochoric Supercooling Organ Preservation System. Bioeng (Basel) 10 10.3390/bioengineering10080934 PMC10451689 37627819

[19] Fahy GM, MacFarlane DR, Angell CA, Meryman HT (1984) Vitrification as an approach to cryopreservation. Cryobiology 21:407–426 6467964 10.1016/0011-2240(84)90079-8

[20] Han Z (2023) Vitrification and nanowarming enable long-term organ cryopreservation and life-sustaining kidney transplantation in a rat model. Nat Commun 14:3407 37296144 10.1038/s41467-023-38824-8 PMC10256770

[21] Fahy GM (2009) Physical and biological aspects of renal vitrification. Organogenesis 5:167–175 20046680 10.4161/org.5.3.9974 PMC2781097

[22] Chiu-Lam A, Staples E, Pepine CJ, Rinaldi C (2021) Perfusion, cryopreservation, and nanowarming of whole hearts using colloidally stable magnetic cryopreservation agent solutions. Sci Adv 7 10.1126/sciadv.abe3005 PMC7793590 33523997

[23] Costanzo JP, do Amaral MC, Rosendale AJ, Lee RE (2013) Hibernation physiology, freezing adaptation and extreme freeze tolerance in a northern population of the wood frog. J Exp Biol 216:3461–3473 23966588 10.1242/jeb.089342

[24] Costanzo JP, Reynolds AM, do Amaral MC, Rosendale AJ, Lee RE (2015) Jr. Cryoprotectants and extreme freeze tolerance in a subarctic population of the wood frog. PLoS ONE 10:e0117234 25688861 10.1371/journal.pone.0117234 PMC4331536

[25] Tessier SN (2022) Partial freezing of rat livers extends preservation time by 5-fold. Nat Commun 13:4008 35840553 10.1038/s41467-022-31490-2 PMC9287450

[26] Tessier SN (2022) The role of antifreeze glycoprotein (AFGP) and polyvinyl alcohol/polyglycerol (X/Z-1000) as ice modulators during partial freezing of rat livers. Front Phys 10 10.3389/fphy.2022.1033613 PMC10161798 37151819

[27] Ozgur OS (2024) Optimized partial freezing protocol enables 10-day storage of rat livers. Sci Rep 14:25260 39448774 10.1038/s41598-024-76674-6 PMC11502795

[28] Goujon JM (1999) Histological evaluation of proximal tubule cell injury in isolated perfused pig kidneys exposed to cold ischemia. J Surg Res 82:228–233 10090834 10.1006/jsre.1998.5526

[29] Matsuno N (2006) Application of machine perfusion preservation as a viability test for marginal kidney graft. Transplantation 82:1425–1428 17164712 10.1097/01.tp.0000243733.77706.99

[30] Meister FA (2020) Decrease of renal resistance during hypothermic oxygenated machine perfusion is associated with early allograft function in extended criteria donation kidney transplantation. Sci Rep 10:17726 33082420 10.1038/s41598-020-74839-7 PMC7575556

[31] Hosgood SA, Barlow AD, Hunter JP, Nicholson ML (2015) Ex vivo normothermic perfusion for quality assessment of marginal donor kidney transplants. Br J Surg 102:1433–1440 26313559 10.1002/bjs.9894

[32] Lyon A (2024) Dietary or pharmacological inhibition of insulin-like growth factor-1 protects from renal ischemia-reperfusion injury in mice. iScience 27:111256 39759002 10.1016/j.isci.2024.111256 PMC11700642

[33] Inker LA (2014) KDOQI US commentary on the 2012 KDIGO clinical practice guideline for the evaluation and management of CKD. Am J Kidney Dis 63:713–735 24647050 10.1053/j.ajkd.2014.01.416

[34] Berkane Y (2023) Supercooling: a promising technique for prolonged preservation in solid organ transplantation, and early perspectives in vascularized composite allografts. Front Transplantation 2 10.3389/frtra.2023.1269706 PMC11052586 38682043

[35] Ozgur OS (2023) Current practice and novel approaches in organ preservation. Front Transpl 2:1156845 10.3389/frtra.2023.1156845 PMC11235303 38993842

[36] Gangwar L (2024) Physical vitrification and nanowarming at human organ scale to enable cryopreservation. bioRxiv

[37] Gt Olverson (2024) Cardiac Loading using Passive Left Atrial Pressurization and Passive Afterload for Graft Assessment. J Vis Exp 10.3791/66624 PMC11815639 39158286

[38] Patel M, Hosgood S, Nicholson ML (2014) The effects of arterial pressure during normothermic kidney perfusion. J Surg Res 191:463–468 24811916 10.1016/j.jss.2014.04.003

[39] Longchamp A (2020) Ex Vivo Analysis of Kidney Graft Viability Using 31P Magnetic Resonance Imaging Spectroscopy. Transplantation 104:1825–1831 32675744 10.1097/TP.0000000000003323

